# A Fatal Case of Legionella bozemanii Pneumonia in an Immunocompetent Patient

**DOI:** 10.1155/2024/7571380

**Published:** 2024-04-02

**Authors:** Atif Saleem Siddiqui

**Affiliations:** Department of Medicine, Division of Pulmonary and Critical Care Medicine, Houston Methodist Hospital, Houston, Texas, USA

## Abstract

Legionella bozemanii pneumonia is a rare form of Legionnaires' disease caused by the bacterium Legionella bozemanii. It is well known to cause pneumonia in immunocompromised patients and has rarely been reported in immunocompetent hosts. We describe a case of a 59-year-old immunocompetent female presented with pneumonia, acute respiratory failure, acute respiratory distress, and septic shock, who was treated with azithromycin, goal-directed resuscitation, and extracorporeal membrane oxygenation (ECMO) but did not survive. Clinicians should have high suspicion of rare legionella pathogens as causative agents for pneumonia.

## 1. Introduction

Legionella bozemanii was first isolated from the lungs of a freshwater scuba diver who died of pneumonia in 1959 [1, 2]. L. bozemanii accounts for 3 to 5% of cases of pneumonia caused by Legionella species [3]. It is a well-known cause of pneumonia in immunocompromised hosts [4–9]. However, it is rarely reported in an immunocompetent host [10]. We describe the case of fatal legionella bozemanii in a previously healthy woman without known medical conditions.

## 2. Case Report

**Figure 1:**
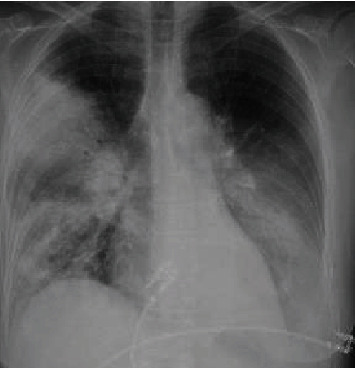
CXR with consolidation in the right upper lobe with patchy airspace opacity in the lower lobe and consolidation in the left lower lobe.

**Figure 2:**
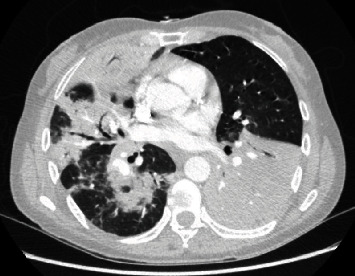
Computed tomography (CT) scan of chest showing consolidation in the left lower lobe, right middle lobe, and right lower lobe with air bronchograms.

**Figure 3:**
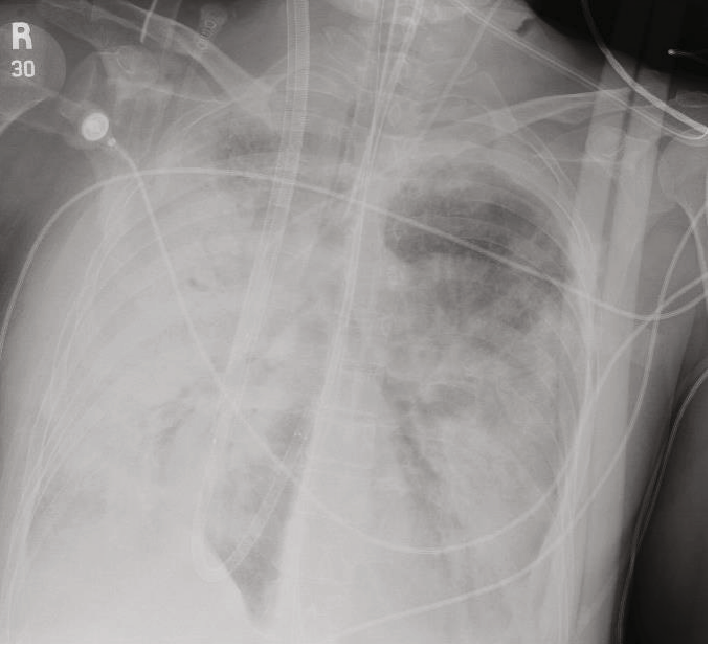
CXR with extensive bilateral consolidation and diffuse bilateral pulmonary infiltrates.

A 59-year-old female with no known past medical history presented to the emergency department with cough, fever, and shortness of breath for 1 week associated with pleuritic chest pain. She denied wheezing, smoking, allergies, or sick contacts. On initial evaluation, her heart rate was 112/min, respiratory rate 24/min, oxygenation saturation 94% on room air, temperature 103.2 F, and normal blood pressure. Chest examination was remarkable for bilateral crackles and diminished breath sounds at the bases. Initial laboratory data showed a white cell count of 12.6 k/UL, hemoglobin 10.7 g/dL, and a platelet count of 206 k/UL. Serum creatinine and serum sodium were normal. Chest X-ray (CXR) and chest-enhanced computed tomography showed multifocal consolidations (Figures 1 and 2). Intravenous antibiotics, ceftriaxone and azithromycin were started on admission. Blood cultures were negative. Urinalysis was normal. Legionella urine antigen test was negative. Urine streptococcus pneumonia antigen test was negative. Extensive immunological workup was negative. The patient's respiratory status rapidly worsened on hospital day 2, and she was intubated. CXR showed diffuse interstitial and alveolar infiltrates (Figure 3). The patient was started on veno-venous extracorporeal membrane oxygenation (ECMO) via femoral-femoral approach with 21 Fr single stage on the right and 25 Fr multistage cannulae on the left. The antibiotics were expanded to vancomycin, cefepime, and azithromycin due to septic shock and multiple pressors were used. She underwent bronchoscopy with bronchoalveolar lavage (BAL). BAL cultures were negative for legionella, acid-fast bacilli, and fungi. Her hospital course was complicated by renal failure and she required continuous renal replacement therapy. She remained in refractory shock despite antibiotics. Repeat blood cultures and repeat BAL remained negative. Her hospital course was further complicated with disseminated intravascular coagulation, and her family decided to change the goals of care to comfort care on hospital day 7. Her family agreed with an autopsy that showed positive cultures for legionella bozemanii from consolidated lung tissue.

## 3. Discussion

The Legionella species are widespread in water and soil environment and can colonize water sources. Legionella bozemanii has also been found in commercial potting soils [11–14]. Legionella bozemanii, a rare cause of pneumonia, is well known to cause impaired cellular immunity and underlying disease but is rare in patients with intact cell-mediated immunity. Sobe et al. described the case of legionella bozemanii pneumonia in an immunocompetent male patient who was diagnosed with video-assisted lung biopsies and treated successfully with erythromycin. Extrapulmonary Legionella bozemanii has been reported to cause soft tissue infections but in immunosuppressed patients [15]. A case associated with prosthetic valve endocarditis in an immunocompetent male has been described in the literature, and the patient was successfully treated with levofloxacin and azithromycin for 6 weeks [16]. The case described in our report is a rare case of legionella bozemanii pneumonia in a young healthy woman. She was not on steroids and other immunosuppressive medications. She did not have underlying lung disease. Interestingly, she had negative bronchoscopy cultures with bronchoalveolar lavage × 2. Her urine legionella antigen test was negative as well. She was treated with appropriate antibiotics including azithromycin for 5 days. Lung biopsies were not performed in this case. She is the first patient with legionella bozemanii pneumonia reported in literature who was treated with ECMO support but still progressed to refractory septic shock, multiorgan failure, and death. The optimal treatment for severe disease includes 5-10 days of azithromycin or levofloxacin [17]. Furthermore, lung biopsies are not the standard of care for bacterial pneumonia. However, clinicians should consider legionella infection if standard bronchoscopy cultures, blood cultures, and urine antigens are unrevealing.

## 4. Conclusion

Clinicians should have high suspicion of rare legionella pathogens as causative agents for pneumonia. Further research is needed to understand its clinical characteristics. The most optimal treatment for severe disease includes five to ten days of levofloxacin or azithromycin. ECMO may be needed as a supportive measure until treatment is successful, or a lung biopsy can be performed safely.
